# *Virk*: an active learning-based system for bootstrapping knowledge base development in the neurosciences

**DOI:** 10.3389/fninf.2013.00038

**Published:** 2013-12-25

**Authors:** Kyle H. Ambert, Aaron M. Cohen, Gully A. P. C. Burns, Eilis Boudreau, Kemal Sonmez

**Affiliations:** ^1^Graph Analytics Operation, Intel LabsBeaverton, OR, USA; ^2^Department of Medical Informatics and Clinical Epidemiology, School of Medicine, Oregon Health and Science UniversityPortland, OR, USA; ^3^School of Engineering, Information Sciences Institute, University of Southern CaliforniaLos Angeles, CA, USA

**Keywords:** active Learning, text-mining, neuroinformatics, biocuration, community-curated database, machine learning

## Abstract

The frequency and volume of newly-published scientific literature is quickly making manual maintenance of publicly-available databases of primary data unrealistic and costly. Although machine learning (ML) can be useful for developing automated approaches to identifying scientific publications containing relevant information for a database, developing such tools necessitates manually annotating an unrealistic number of documents. One approach to this problem, active learning (AL), builds classification models by iteratively identifying documents that provide the most information to a classifier. Although this approach has been shown to be effective for related problems, in the context of scientific databases curation, it falls short. We present *Virk*, an AL system that, while being trained, simultaneously learns a classification model and identifies documents having information of interest for a knowledge base. Our approach uses a support vector machine (SVM) classifier with input features derived from neuroscience-related publications from the primary literature. Using our approach, we were able to increase the size of the Neuron Registry, a knowledge base of neuron-related information, by a factor of 90%, a knowledge base of neuron-related information, in 3 months. Using standard biocuration methods, it would have taken between 1 and 2 years to make the same number of contributions to the Neuron Registry. Here, we describe the system pipeline in detail, and evaluate its performance against other approaches to sampling in AL.

## 1. Introduction

In 2008, Howe and colleagues proposed that in the next 5 years the field of biocuration should create a mechanism by which community-based curation efforts can be facilitated (Howe et al., 2008). This challenge has been taken on and applied in already successful online databases, such as FlyBase^1^, the online repository of drosophila genetic information, and GrainGenes^2^, a browser for the *triticeae* and *avena* genomes, but is not so easily translated to new databases and knowledge bases, which neither have an active contributing community, nor are able to support the professional staff to maintain them. As the neuroinformatics community begins to rely more on online resources containing structured information that can be used for large-scale mathematical modeling and simulation, the ability to efficiently create a new database and expand it to the point that it is a useful resource to the research community will increase in importance, even as the amount of information that must be manually searched through increases with the increasing rates of publication. It is because of this that the community must turn to automated techniques that have demonstrated their effectiveness in the field of machine learning (ML). Such techniques, in the ML community, are known as recommender systems (Resnick and Varian, 1997).

The process of manually curating data from published papers into computationally-accessible databases is an important (and mostly unacknowledged) bottleneck for developers of neuroinformatics resources. For example, the first version of the CoCoMac system [*Collations of Connectivity data on the Macaque brain*^3^ (Stephan et al., 2001)] is a neuroinformatics database project concerned with inter-area connections in the cerebral cortex of the Macaque. It is a mature solution for a problem that was under consideration by national committees as far back as 1989 (L.W. Swanson, personal communication). CoCoMac currently contains roughly 2.0 × 10^4^ connection reports, reflecting the dedicated effort of a small curation team over years of work. Due to the machine-readable nature of much of the data in their field, bioinformatics systems in molecular biology are usually larger by *several orders of magnitude*. The Uniprot KB release for February 2013, for example, contains 3.03 × 10^7^ entries. Naturally, this difference is due to many factors, including the levels of available resources for curation, the general utility of the data being housed, and the relative size of user communities. In any case, the rate-determining step for developing knowledge bases in any domain of application is the speed of curation. Thus, accelerating that process is an central goal to the data curation community.

To mitigate the problems associated with an increased need for curated information, biocurators should adopt automated approaches to identifying documents containing information relevant to their particular knowledge base, such as active learning (AL) systems. An extensive review of AL methods is available from Burr Settles, (Settles, 2010) but, briefly, AL is a type of supervised ML framework, in which a classification algorithm works collaboratively with an expert user to efficiently train a classification model (in terms of the expert's effort), by requesting expert labels for the data (also known as gold-standard annotations) for the data the AL system deems most informative. Such methods could be incredibly useful for neuroinformaticians starting up new knowledge bases, helping them to efficiently create, for example, a manuscript recommendation system that would allow for spending more time reviewing manuscripts that are likely to contain information of interest.

A substantial body of work has demonstrated the effectiveness of AL and recommender systems for efficiently developing document classifiers that have been incrementally trained on gold-standard data. Mohamed and colleagues, for example, used AL to develop a protein–protein interaction predictor (Mohamed et al., 2010), and Arens, in conjunction with a support vector machine (SVM) classifier, used AL for learning document ranking functions in a biomedical information retrieval task (Arens, 2010). SVMs and AL have also been paired together for the identification of documents that are eligible for inclusion in a systematic review (Wallace et al., 2010). Here, Wallace and colleagues adapted previously-developed AL strategies for biomedical document classification by taking into account the commonly-observed highly-skewed class distribution in such publications.

The work presented here describes an AL-based approach to rapidly developing specialized knowledge bases deriving their information from scientific publications, all while simultaneously training a classification model that can be used to identify new documents, as in a traditional supervised document classification-type framework. Our workflow involved a few simple modifications of the traditional AL workflow, incorporating the steps needed to identify publications of interest within a corpus without corrupting the AL classification model by giving it a false picture of the class distribution in the data set. Our procedure can be easily adopted by informaticians lacking the labeled training data necessary to train a classifier who wish to use ML to develop a knowledge base. Because of the International Neuroinformatics Coordinating Facility's (INCF) emphasis of developing computationally-accessible resources that can be used in multi-level models of neuroscientific data (Cannon et al., 2007), to evaluate our system we focused our efforts on an under-populated knowledge base containing neuron-related information extracted from the neuroscience literaturebase.

The Neuron Registry (NR) is a community-curated knowledge base under the direction of the Neuron Registry Task Force (NRTF^4^), a part of the INCF Program on Ontologies of Neural Structures (PONS). The primary goal of the NRTF is to create the infrastructure for a machine-readable knowledge base of neuronal cell types, providing a formal means for describing and quantifying existing cell types from their properties, and populating it with information that has been extracted from the primary literature. It's curator interface can be accessed at^5^.

As a community-curated knowledge base, growth of the NR is contingent upon user submissions—the problem of adding new information to the system has been largely left to the people who use it. For knowledge bases that already have a strong user-base and an active community (e.g., Wikipedia), new submissions are frequently being made. This makes sense—Wikipedia is one of the more frequently-accessed web sites in the world; for a less-well-known resource, such as the NR (which has contributions from only 13 individuals, to date), some level of usefulness will need to be demonstrated before it becomes a resource to which researchers are regularly willing to submit new information (Burge et al., 2012). Given the broad scope of information that is relevant to the NR, a great many more contributions will need to be made before it can be used as a reliable, repository of the community's neuron-related knowledge. This is a common problem in informatics, in general, and neuroinformatics in particular. New web-based resources are frequently created and made publicly available for use in others' research. Initially, the creation and maintenance of such resources is often supported by the grant that lead to their creation, but it is uncommon for funds to be available for the continued maintenance of a resource that has not already demonstrated meaningful contributions to the research community (Ambert and Cohen, 2012). This can lead to the gradual decline of a resource, to the point where it is no longer a reliable, up-to-date snapshot of the community's knowledge. For example, this happened with the well-known CoCoMac database, which was unable to keep up with the pace of the published literature on Macaque connectivity beyond 2008, because of increasing rates of publication and limited resources (Kötter, personal communication; 2009). Thus, informaticians interested in creating accessible knowledge bases for the research community are left with a dilemma: how can they create a resource and deploy it with sufficient information, without spending a great deal of time and money on curating the information they wish to include before the user community has been established? From the ML community, the answer to such a problem has been AL and recommender systems.

Although AL has been shown to be useful for identifying documents that will provide the most information to a supervised classification system, no one has yet used AL for simultaneously identifying new documents containing relevant information for a knowledge base while training a new document classifier for later use in updating the knowledge base. Although the relatively new field of ontology learning addresses a similar issue [for a review, see Subramaniyaswamy et al. (2013) and Wong et al. (2012)], by simultaneously creating an ontology while the learning process is going on, this approach has yet to be adopted in the realm of AL for document classification. While the creation of a data set for training document classifiers is useful to the biocuration community (for a review, see Hirschman et al., 2012), it should also be possible to use AL to streamline the process of identifying an initial set of documents containing information of interest to under-developed knowledge bases lacking sufficient data for training a classifier. There are two main hurdles to achieving this goal. First, a system needs to simultaneously identify documents that are likely to contain information of interest while identifying documents on which it cannot reliably make a judgment. Second, the existing methods for evaluating AL systems have been designed to work with a large, fixed corpus of data already annotated, which is not available for our purposes—no annotated full text corpus of neuron-related documents has been made available to the public. What's more, existing evaluation metrics primarily focus on the accuracy of the classifier being built and the rate at which it was able to achieve peak performance (Settles, 2010). While these aspects of the proposed system are important, we are also interested in the point at which the maximum number of relevant documents are identified at the minimum amount of annotation effort—this trade-off does not exist in typical AL applications.

Here, we present and evaluate *Virk*, an AL system that is able to rapidly bootstrap knowledge base development. Over the course of our experiments, we dramatically increase the coverage of the NR, which will make it possible for the knowledge base to be a more useful resource to neuroscientists, and to create a unique, publicly-available expert-curated document collection for the neurosciences that will be useful to neuroscientists and text-mining researchers in the future. We describe our gold-standard-trained recommender system that was used to contribute to the NR, demonstrating, for the first time, that, with minimal effort spent on tuning a classification system, an AL system can provide meaningful contributions to the biocuration workflow. Importantly, our system is designed to specifically address bottlenecks in the NR curation workflow. As a tool primarily designed for bootstrapping the start-up of new knowledge bases, the Virk system will help developers deal with the significant volume of publication they must review, but for which they may have no prior information that can help them efficiently prioritize how they should do their reading. Virk will help biocurators by interactively re-ordering a list of publications in terms of their likely relevance, updating its judgments after receiving feedback from the curation staff.

## 2. Materials and methods

### 2.1. Collecting a full text neuron-related document set

In practice, the procedure used to collect a document corpus is going to differ from use case to use case. In some applications (e.g., systematic review), the user may already have a large collection of unlabeled documents (Ananiadou et al., 2009; Cohen et al., 2010a). In other applications, it may not be necessary to ensure that a document collection that is narrow in focus is obtained. However, because, in this study, we are interested in evaluating the AL process for a neuron-related knowledge base, and we did not have any full text publications available to us ahead of time, we manually acquired our corpus. Data collection proceeded in two main stages: journal selection, and article selection. First, we determined which neuroscience-related journals to use to build our document corpus. Our primary goal was to build a document collection adequately representing the diversity of the neuroscience literature, so that our classifier would be exposed to articles reflecting the diversity of neuroscience (e.g., neuroimaging, computational neuroscience, and behavior), in addition to documents specifically containing information on neuron-related experiments. At the same time, we wanted to obtain a sufficient number of documents containing information relevant to the NR. Thus, we downloaded the all entries in *vertebrate neuron* category on NeuroLex^6^, an online, community-curated neuroscience lexicon, and found which journals most often include these terms within the MEDLINE records of the research articles they publish. We wanted to be certain that the articles we eventually included in our corpus had complete MEDLINE records and were representative of the sorts of terminology used in newly-published research, so we decided to limit our document selection to those published during the year 2010.

PubMed^7^ queries were constructed for each selected NeuroLex term, each taking the form “NEURON,” where NEURON corresponded to an entry in the NeuroLex (e.g., “*Amygdala basolateral nuclear complex pyramidal neuron*”). We rank-ordered the journals, in terms of their frequency of using NeuroLex terms in 2010. The top eight journals are listed in Table 1. We limited our selected journals to eight, because the ninth journal was the *Proceeds of the National Academy of Science*, which would have doubled the size of our corpus and would have also diluted the concentration of annotatable information by adding many non-neuroscience articles (e.g., geology or astronomy).Column *n*_2010_ in Table 1 shows the number of publications associated with that particular journal in 2010. The complete MEDLINE records for all 5932 articles included in Table 1 were downloaded and stored in a mongoDB database^8^. We chose a document-oriented database for this work because they allow us to efficiently represent MEDLINE records, which often have fields that differ from document to document. Since our University library had subscriptions to the eight journals of interest, we attempted to download the full text for each. Due to time constraints, we only attempted to download each of the 5932 full text documents once—if an error occurred, we simply skipped that document. In all, we were able to successfully download 3336 of the documents we were interested in. Since the approximately 2500 articles we were unable to download were distributed amongst over six journals representative of the neuroscience literature, and 3336 was likely to be an adequately-sized data set, no further attempt to retrieve these articles was made.

**Table 1:** **Table of PubMed results by the top nine-matching journals in the data set, based on performing queries with the NeuroLex terms**.

**Journal**	***n*_2010_**	**Prevalence (%)**
The Journal of Neuroscience	1457	26.4
Brain Research	1267	19.9
Neuroscience Letters	1056	27.3
Neuroscience	910	NA
The Journal of Physiology	495	14.4
The Journal of Comparative Neurology	276	NA
Hearing Research	256	6.1
Journal of Neurophysiology	215	5.8
Total	5932

*n_2010_ denotes the number of 2010 publications associated with that journal. The prevalence column denotes the percent representation of that journal in the final data set. Two journals—Neuroscience, and The Journal of Comparative Neurology, are not represented in the final data set, as we were unable to download their articles*.

**Figure 1:**
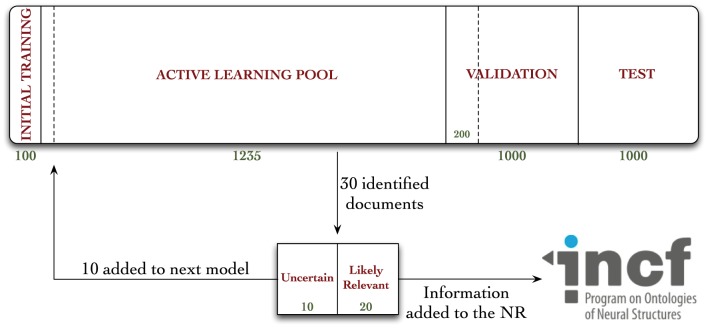
**Diagrammatic representation of the distribution of documents in our corpus, and workflow diagram for annotation in the active learning experiment**. Documents were randomly allocated to either the initial training, active learning pool, validation, or test collections. A total of 962 documents were annotated during these experiments—670 during the active learning procedure, 92 during the random validation experiments, and an additional 200 for the hold-out test collection, used to evaluate the Virk system against the random system.

The 3336 documents were associated with their respective MEDLINE records in our database, and distributed into one of four document pools, according to Figure 1.

### 2.2. Procedure for annotating the documents

One of the goals of this study was to build up a document classification data set for identifying publications likely to contain relevant information for the NR. The NR is a collection of neuron-related information, in the form of rows of (neuron type, relation, value) tuples (e.g., *CA1 pyramidal cell*, *located in*, *CA1 stratum oriens*), and an associated reference (e.g., a PubMed identifier). Previous work has shown that having well-defined annotation schema and criteria is important for building up a consistent document collection for ML. Thus, in collaboration with Giorgio Ascoli at the Neuron Registry, we developed an annotation schema where an article was marked *excluded* unless it could meet the following inclusion criteria:

1. The document appears in a peer-reviewed scientific journal.
2. The document is a primary source of the information in question (i.e., a primary, citable communication of the information in question (and not, for example, a review article).
3. The document contains all parts of the (Neuron Type, Relation, Value, Publication ID) tuple.
4. The Neuron Type and Relation identified in the document in question are found in the accepted set of values:
  - The Neuron Type must either map to one of the types listed on neurolex.org^9^, or, if it's not included, a strong case must be able to made for needing to include it.
  - The relation must be an accepted NR relation type (see Table 2).
5. The (Neuron Type, Relation, Value, Publication ID) tuple must not already be included in the NR.

**Table 2:** **The list of accepted Neuron Registry neuron relation values used for creating the annotated document collection**.

**Accepted Neuron Registry neuron relations**	
Expresses protein	Does not express protein
Has molecule	Does not have molecule
Makes contact to	Does not make contact to
Receives contact from	Does not receive contact from
Located in	Not located in
Has current	Has firing pattern
Has part	Lacks part
Has orientation	Generates
Has mRNA transcript	Expresses gene
Lacks quality	Has quality
Has size	Has shape

*The list was generated by examining entries already annotated into the neuron registry, and by manual examination of neuroscience publications*.

During the process described below, documents were annotated for inclusion in the NR, and, if the document contained information relevant to the NR, the specific text which led to this judgment was extracted and the information was uploaded to the NR website.

### 2.3. Developing and training the baseline classifier

Hundred randomly-selected documents were annotated for the Initial Training collection (Figure 1). We used these documents to conduct a set of classification experiments that would help us determine the feature types and modeling approaches used by the AL classifier. Our intention was to create a classifier that could make accurate classification judgements without using many enriched-engineered features (e.g., named entity recognition, or part of speech tagging). We made an *a priori* decision to use a SVM classifier with linear kernel (Fan et al., 2008), using default parameter settings, as we have used this for a baseline classifier in previous work (Cohen, 2008; Ambert and Cohen, 2009, 2011; Cohen et al., 2009, 2010c). Our goal in developing the baseline classifier was not to create a highly-optimized classifier tuned to work on neuroscience publications. Rather, we aimed to identify, from amongst a set of simple feature types and feature modeling approaches, a system that performed well on the initial training corpus (e.g., an area under the receiver-operator curve—or AUC—greater than 0.75), and which was not complicate—both performance and parsimony motivated our decision. To this end, our choices were informed by simple comparison of AUCs, not statistical analysis of the results presented in Table 3.

**Table 3:**
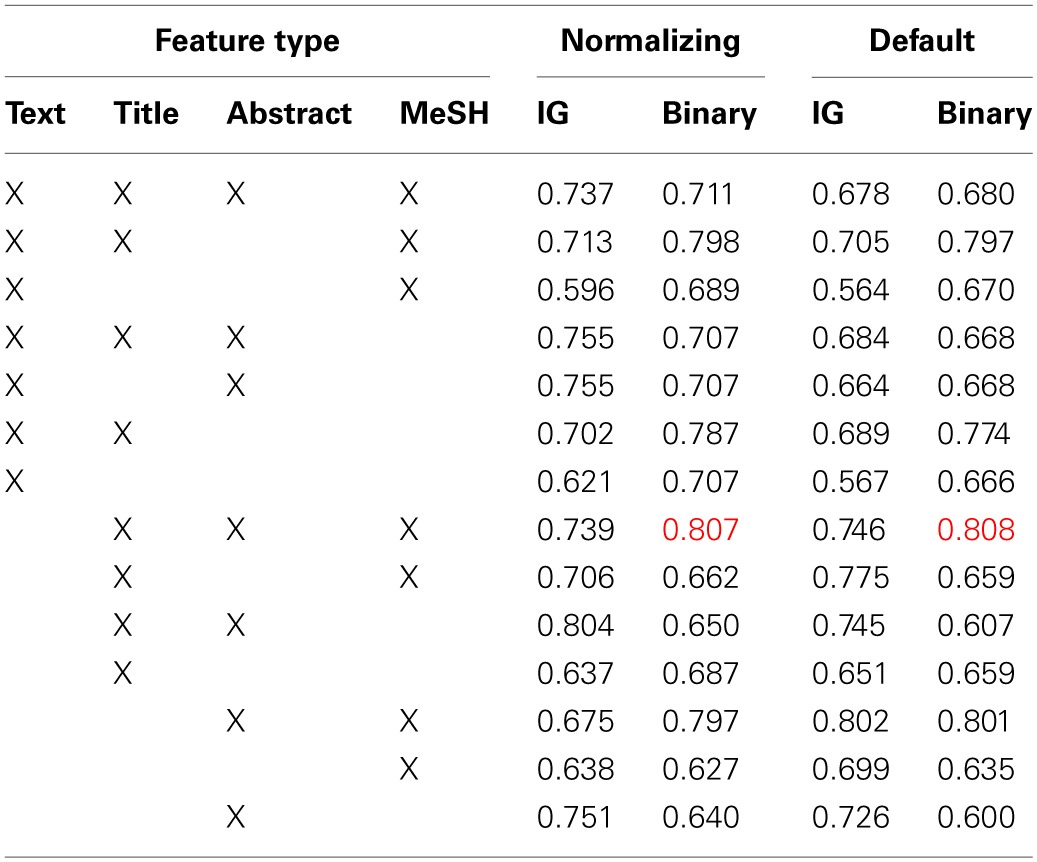
**Summary of area under the ROC curve (AUC) results observed in the baseline classifier cross-validation (five repetitions of two-way; 5 × 2) experiments**.

*An X in any of the four left columns indicates the inclusion of textual features from that portion of the document. The four right-hand columns show the AUC observed when using either the Normalizing Feature Combiner (columns five and six), or the Default Feature Combiner (columns seven and eight), along with either the Information Gain-based (IG) or binary modelers. The AUCs highlighted in red denote the two top-scoring system configurations. The top-performing baseline systems were both obtained from systems using unigrams derived from documents' title, abstract, and MeSH terms*.

Another motivation for the present experiments was to create a document corpus that could be used for supervised document classification experiments down the road. If a more complex classifier using engineered features were used here, it's possible that the selections it makes could affect the results of those future experiments. Thus, to focus on the AL process in our work, we aimed to use the simplest possible classification pipeline. We decided there were three aspects of our classification system we could optimize for the baseline classifier: input features, feature normalization, and modeling type. The input features we investigated were all combinations of Medical Subject Headings (MeSH terms), abstract unigrams, and title unigrams (as obtained from the associated MEDLINE record), and full text unigrams (as obtained from the above-described pdf-to-text extraction procedure). No other fields in the MEDLINE record were used in this study, however, if any of that information appears in the full text (e.g., author names), that information would be represented in the full text feature set. In our approach, we define unigrams as individual space- or punctuation-separated words. We intentionally leave the question of whether more complex feature types (e.g., bigrams, or named entity recognition-derived features) to future work, once a corpus has been created. We considered two feature vector normalization techniques—one in which the features from different sections of the document are simply combined into a single larger vector of greater dimension, and another which applies L2 normalization to the vector components:

(1) $$|x|=\sqrt{\sum_{k=1}^{n}|x_{k}{|}^{2}},$$

where **x** is a vector of length *n*. Finally, we considered two feature modeling methods: binary, and mutual information-based. In binary feature modeling, each document in the training collection is represented as an *n*-length vector of 0' s and 1' s, where *n* corresponds to the unique set of features in the training collection, and a given index in each document's vector maps to the same feature. When applying the training model to unseen data, then, only the features that were observed in the training collection are used to make the classification judgment—any previously unseen features are ignored. Mutual Information-based feature modeling is similar, however, the role of each index of the vector in this method corresponds to the mutual information of that feature for distinguishing between the two classes, where we define mutual information, or information gain, of feature *j* in all documents as

(2) $$IG_{x_{.j}}=H(x_{.j})-H(x_{.j}|y_{i}),$$

where *x* is a feature matrix with dimensions *i* (number documents) x *j* (number of features), . denotes performing some operation over all elements of that dimension, and *y*_*i*_ corresponds to the true class label *y* of document *i*, and *H*(*x*_.*j*_) is the entropy of feature *j* in the collection of documents, defined as

(3) $$H(x_{.j})=-\sum p(x_{.j})\log p(x_{.j})$$

The results from our baseline classifier experiments are shown in Table 3. As is shown there, the two top-performing systems, in terms of the area under the ROC curve (AUC), a standard measure of performance for a binary classifier, observed on five repetitions of two-way cross-validation (5 × 2 cross-validation; a method for assessing the performance of a classification system) using the 100 manually-annotated documents from the Initial Training collection (Figure 1), used features from the title, abstract, and MeSH terms from each paper, and binary feature modeling using either the default or normalizing feature combiners. We intentionally selected 5 × 2 cross-validation, as we have observed int he past that this approach is less affected by difference in class prevalence (Cohen et al., 2009; Ambert and Cohen, 2011). The default mode for combining features involved simply combining all features into a binary vector, while the normalization approach involves also normalizing the length of the vector to 1. Since the difference between the two top-performing systems was small (0.001), we opted to use the simpler of the two—the one using default feature combining—for our AL experiments.

### 2.4. The active learning procedure

We had two simultaneous goals with our AL system: to learn more about the efficiency of our ML approach in the domain of neuron-related documents, and to identify the greatest number of annotatable documents for the NR as possible, in the least amount of time, and with the fewest-possible total documents examined. In a typical AL text-mining experiment, ML scientists will use a corpus of documents that has already been annotated for a particular task [e.g., the Reuters Corpus, as in Liere and Tadepalli (1997); McCallum and Nigam (1998); Schohn and Cohn (2000); Tong and Koller (2002); Bordes et al. (2005); and Ertekin et al. (2007)]. This is because such studies are concerned with coming to a greater understanding of what classification approaches, modeling techniques, and input feature types lead to best classifier performance in an AL framework. Here, as is often the case with an under-developed or new knowledge base, we have no gold standard available to us. If such a corpus were available, we would simply train a document classifier using the data available, and use the classifier to identify newly-published documents that contain information relevant to the NR. Although it would be possible to create a classifier using the little data already in the NR, it results in a classifier trained to identify documents containing only a small set of neuron-related concepts (e.g., only documents containing, for example, the word *purkinje cell*). This is because, when only a small amount of data is available for a very broad and terminologically-rich field, such as neuroscience, there is not enough information available to create a general representation of the sorts of documents that one is interested in. Often times, the small amount of data available may be about a small set of concepts for which there has been a great deal of research (e.g., *purkinje cells*), making mentions of specific neuroanatomical features highly-predictive terms for a classifier built to identify documents containing information that is similar to those already included in the knowledge base. Ideally, a classifier would make its judgments based on more general concepts, such as methods that are often used in NR-relevant publications (e.g., patch-clamp), or observation-related words associated with those types of methods (e.g., current), but, to learn these types of associations, the classifier would need to be presented with many more examples of relevant and irrelevant documents than were available to the NR, (or, indeed, than often are available to many new knowledge bases). Here we create a method for using AL to bootstrap the development of a knowledge base while simultaneously training a document classifier. To our knowledge, this is the first-published method accomplishing this task.

A workflow diagram of our annotation procedure can be seen in Figure 1. We trained our baseline classifier on the annotated Initial Training sample, and classified all 1235 of the documents in the AL pool. We rank-ordered these judgments in terms of confidence, where a confidence of 1.0 is a document that our system is highly confident is one containing annotatable information, to 0.0, which is one the system is least confident the document contains annotatable information (or, most confident that it does not contain such information). There are a variety of AL sample selection methods that have been used in previous work (for a review, see Settles, 2010). We chose this approach for its simplicity and efficiency. From the rank-ordered list, we identified 30 documents—the top 20 highest confidence that were most likely to contain annotatable information, and the 10 that the classifier was least certain about (i.e., the 10 nearest to a confidence value of 0.5). These numbers of documents were selected because we thought they would provide the right balance of possible granularity in our performance metrics, while still being small enough that we could detect changes in classifier performance. All articles were read in full, and annotated as positive or negative for containing information relevant to the NR (the terms positive-class/relevant and negative-class/not relevant are used interchangeably in this manuscript). For those found to contain annotatable information, the relevant data was manually extracted and uploaded to the NR. The annotated 10 uncertain documents were then added to the documents from the Initial Training sample (giving 110 annotated documents), the model was re-trained, and the remaining documents in the AL pool were re-classified (already-annotated documents are removed from the AL pool). Importantly, any features observed in the new documents that were not already observed over previous iterations were added to the classification model. The whole process was repeated for 20 iterations. At each iteration, we encounter both positive- and negative-class documents. Both classes of documents are included in the final data set that we have released for use in text-mining and neuroinformatics research. We chose to only include the 10 uncertain documents, rather than using all 30 annotated at each iteration, in the data the model was trained on because we hypothesized that, while adding all 30 documents would likely help boost the model's performance, in terms of AUC, it wouldn't necessarily help us pick the most useful documents for classifier training, and bias the classifier toward positive prediction (see Discussion section).

This procedure highlights the dual-purpose of this set of experiments: the 10 uncertain documents are akin to those that would be added at each iteration of a typical AL experiment, while the 20 most likely relevant documents are identified at each iteration of our procedure so that we are more likely to identify documents containing actual annotatable information at each iteration.

### 2.5. Evaluating the dual-purpose approach to using active learning

We evaluated our general approach in terms of the change in classifier performance over AL iterations, and the change in the ratio of the ML-predicted relevant to truly relevant documents. The former relates to the performance metrics often used in other AL and text classification studies—change in AUC over learning iterations. We chose AUC as a performance metric because we were primarily interested in our system producing accurate rank-orderings, rather than completely accurate predictions—that is, it was more important to us that the top 20 documents that were predicted to contain annotatable information actually contained such information, than whether or not the SVM actually predicted them as belonging to the positive class. The later performance metric has to do with our goal of developing an AL system that is able to bootstrap knowledge base development by identifying publications that are likely to be relevant. We would expect that, if our system is able to accomplish this task, the number of truly relevant publications that it identifies would increase during the initial stages of training, level out for a time, and then begin to decline again, once the relevant documents in the AL pool collection become more rare.

In order to determine whether the classifier would be better off being trained by all 30 manually-annotated documents at each iteration, rather than just the 10 uncertain documents, we ran a set of experiments comparing the two possible approaches. To do this, we annotated an additional 200 randomly-selected documents from the hold-out validation pool. We trained a classifier using the documents that were selected up to each iteration in the original run of AL experiments, classifying the 200 validation documents using one of two methods—either a model which was trained using only the 10 uncertain documents identified at each iteration, or using a model trained on all 30 documents (the 10 uncertain documents, and the 20 predicted relevant ones).

Finally, we wished to compare the performance of our system against a randomly-performing system not using an AL document selection mechanism. To do this, we started a new AL system from the same 100 documents selected to initialize the Virk system, and then randomly selected 10 documents from the AL document pool over 19 sampling iterations. If a selected document had not previously been annotated, the document was annotated, and, if it was a positive-class document, it was used to add one or more annotations to the NR. To simulate the process of random sampling over 190 iterations (10 rounds of 19 iterations each), we randomly assigned each of these documents to one iteration in each round. At each iteration of both systems, the systems were trained on their annotated training data and evaluated against 200 randomly selected documents from the previously-described 200 documents that were annotated from the hold-out validation collection.

## 3. Results

Hundred randomly-selected documents were annotated for the first iteration of training, of which eight were manually determined to contain NR-relevant information. Based on this, we inferred an 8% positive sample inclusion rate (4.4–14.8%, 90% CI, based on the binomial distribution) in the larger population of potentially-included documents.

**Figure 2:**
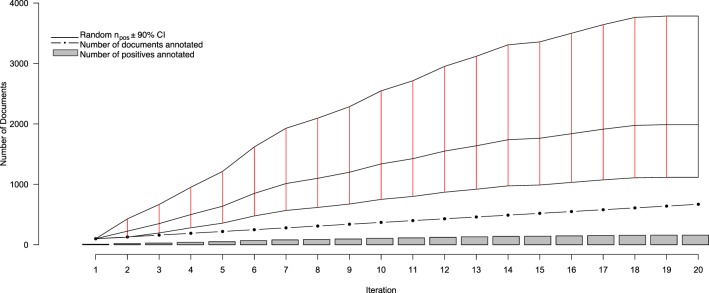
**Performance statistics for the active learning system over iterations of document curation**. The gray bars show the cumulating number of positively-annotated documents, while the black dotted line indicate the total number of documents annotated at a given iteration (increasing by 30 at each iteration after the first). The solid black line intersected with solid red lines indicates the estimated number of randomly-selected documents (±90% CI) that, at any iteration, would need to be annotated in order to obtain the same number of positive documents identified by Virk by that iteration. After three rounds of annotation, the average number of documents that would have to be read for a given number of positives is statistically significantly greater than that needing to be annotated with the Virk system.

Over 20 iterations of AL, a total of 670 full text documents were annotated, over the course of 4 months, for containing information to include in the NR. Of those, 159 were identified for inclusion, with the remaining 511 being excluded. Thus, after 20 iteration we observed a positive inclusion rate of approximately 24%—well outside the originally-projected inclusion rate of between 4.4 and 14.8%. This, of course, is to be expected from a system designed, in part, to identify positive-class documents. Figure 2 depicts the progress of our annotation and AL procedure. From this figure, it is clear that there are different perspectives from which one can assess performance of our system—such as the rate at which positives are identified by Virk, and the savings conferred by Virk over the random system. One consequence of the system identifying a finite number of relevant documents from a fixed pool is that, as the number of positive-class documents in the pool depletes, the classification task becomes more difficult as the positive documents become more rare. To account for this, we evaluated our system in terms of an adjusted positive inclusion rate, defined as

(4) $$AIR=\frac{\frac{n_{\text{pos20}}}{20}}{\frac{{\hat{n}}_{\text{posRemaining}}}{n_{\text{Remaining}}}},$$

where *n*_pos20_ is the number of positive-class documents identified in a round of classification, and ${\hat{n}}_{\text{posRemaining}}$ is the number of positives estimated to remain in the AL pool, based on the initially-estimated positive prevalence rate, and *n*_Remaining_ is the number of documents remaining in the AL pool. This metric will adjust the fraction of positive-class documents found during one iteration of AL by the number of positive-class documents that are estimated to remain in the AL pool, thus accounting for the change in difficulty of the task at each iteration.

**Figure 3:**
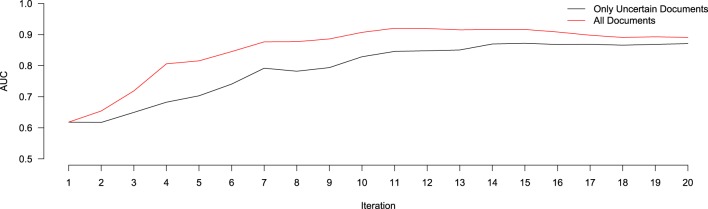
**Relative performance of a system trained using only the uncertain documents at each iteration of active learning (black), versus all documents annotated up to that point (red), in terms of AUC**. At Iteration 1, both systems are trained using the same data (the 100 initially-annotated documents), and thus score the same. After that, the system trained using all the data consistently out-performs the one using only the uncertain data, though both begin to converge to similar values after 20 iterations.

In order to evaluate the effect of only using the 10 uncertain documents to train our classifier (as opposed to all 30 annotated), we ran an experiment to compare the relative performance (in terms of AUC) of a system classifying a hold-out validation set of 200 documents using either a model trained on just the uncertain documents at each iteration, versus one trained on all annotated documents at each iteration. Of these 200 documents, 28 were found to contain relevant information for the NR, while the remaining 172 did not. This resulted in a positive-prevalence rate of 14%, which is within the 90% confidence interval of the original estimated positive-prevalence rate conducted at the outset of the study. The results of this experiment are shown in Figure 3. In terms of AUC, the system trained using the all annotated documents consistently out-performs the one trained using only the 10 uncertain documents at each iteration, though both systems begin to converge to similar values after 20 iterations. This implies that, despite the fact that the classifier is getting trained on a corpus of documents with a class-distribution different from that of the larger population, the extra information contained in the additional documents improved the ranking performance of the classifier. Importantly, however, this does not reflect the impact that this change in training samples might have on our ability to identify the most informative documents for subsequent training, based on the most uncertain predictions of the classifier. With the lowest-confidence sample selection method that we used here, including the 20 most confident documents in the training would have raised the proportion of positive samples in the training data. This likely would have biased the confidence estimates upward, leading to the system being trained with more negative documents and fewer positives. We will return to this issue in the Discussion section. Prior to beginning this study, there were 235 entries in the NR, derived from 16 different journals, and submitted by 13 different authors. The NR development team added the majority of these submissions between April 2010, and April 2011. Over the course of 4 months of annotation using Virk, an additional 257 annotations were added to the NR (90% of the number of entries that it included prior to our work). This expanded the NR coverage of NeuroLex^10^ neuron types from 16 to 55%. Using the class prevalence derived from our initial sample, using a random-selection approach, one would need to review between 160 and 570% more documents than our approach required (between 1116 and 3785 total documents).

To examine the validity of our selection approach and performance metrics, we compared the Virk system to that of a system using the data selected for the first iteration of Virk, and 10 randomly-selected documents at each of 19 iterations (if the document had not already been annotated during the Virk process, it was annotated and added to the NR, if necessary), using the system to classify the 200 hold-out validation documents every iteration. The random process was repeated 10 times, so that we could calculate confidence intervals.

**Figure 4:**
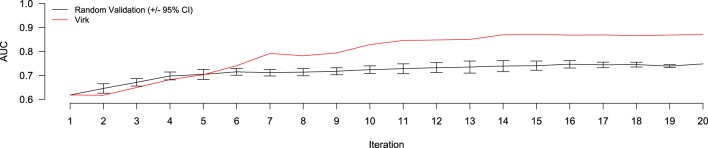
**Performance evaluation comparing AUC of the Virk (red line) and Random Validation (black line) systems over iterations of active learning**. The Random Validation system AUC was averaged over 10 random samplings, so that standard error could be calculated (bars). After six iteration, the Virk system outperform the 95% confidence interval for the random validation system.

**Figure 5:**
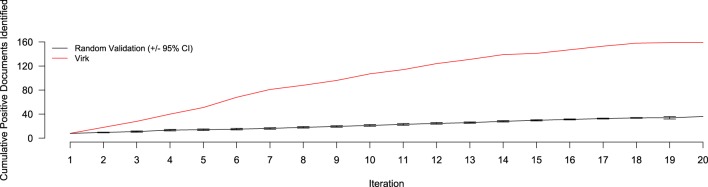
**Number of positive-class documents identified over 20 iterations by Virk (red line) and Random Validation (black line, ±95% confidence interval)**. After 20 iterations, the random validation system identified the number of positives found after only three iterations of the Virk system.

The results of the random validation experiment are shown in Figures 4, 5. To compare the Virk and Random Validation systems, we used area under the AUC obtained from training on the data available at a particular iteration, and classifying the hold-out validation set of 200 documents that were randomly selected from the Validation partition of the data set. As can be seen in Figure 4, for the first five iteration, the Virk system performs worse than random, although the performance differences are not substantially significant. By the sixth iteration, performance of the Virk system greatly exceeds that of the Random Validation system. Peak performance by Virk (AUC ≈ 0.87) appears to occur around iteration 14, while peak performance of Random Validation levels out by iteration 12 (AUC ≈ 0.72). Figure 5 compares the number of positive-class documents identified by the Virk and Random Validation systems. After 20 iterations, Virk identified 159 documents containing information relevant to the NR, whereas the Random Validation system only identified an average of 36 documents. After 4 iterations, our system exceeded the average number of positive-class documents identified using 20 iterations of random sampling. On average, the Random Validation system was able to identify approximately 1.5 positive-class documents per iteration (compared to approximately 8.0, by Virk). Thus, one would have to complete 106 iterations of annotation by random sampling to achieve what our approach was able to do in 20—a greater than 500% difference in work savings. These results demonstrate that Virk is able to quickly out-perform the standard document identification approaches used by biocurators today—our approach was able to identify significantly more relevant documents than the standard approach, and was able to do so with significantly less annotation effort.

**Figure 6:**
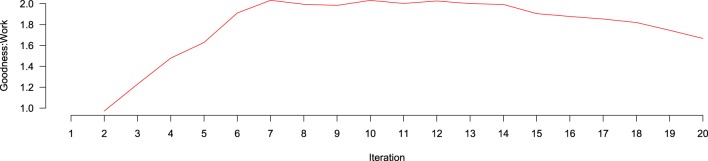
**Goodness: work ratio over iterations of active learning**. No data exists at the first iteration because no active learning has yet taken place. Between iterations 2 and 7, the goodness:work ratio increases, being approximately level until iteration 15, where it begins to decline.

**Figure 7:**
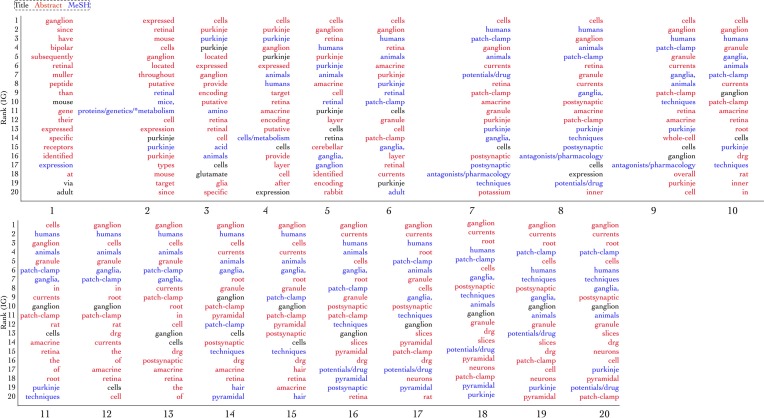
**The top 20 rank-ordered features, in terms of information gain, over iterations of active learning**. The color of the text denotes which section of the document's associated MEDLINE record the term came from: either title (black), abstract (red), or MeSH (blue). Certain terms, such as ganglion are found across many iterations, though its position in the rank-ordered list changes, while others, such as “via” were less informative, appearing only in the first iteration.

While performance metrics such as AUC and number of positive documents identified are important to assessing a classification system, in the case of AL, they do not necessarily tell the whole story. Besides being able to establish that our system can make accurate classifications, we also wanted to understand the trade-off annotators must make when deciding whether to use the system developed at any particular point or make additional annotations. To address this, we developed a metric called *goodness:work*, which quantifies the level of benefit obtained from accurate classifications made by the system relative to the amount of work that has been done in developing it up to that point. Biocurators could use a measure such as this to make informed judgments about when to stop data curation to train an AL system, or to make informed decisions about how many documents would need to be annotated for future related systems. We define the goodness:work measure at iteration *i* as

(5) $${\left(g:w\right)}_{i}=\frac{n_{\text{positives identified}}/n_{\text{total positives estimated}}}{n_{\text{annotated document}}/n_{\text{documents in AL pool}}}$$

goodness:work over iterations is depicted in Figure 6. goodness:work steadily increases, until it is maximized around iteration 7, where it remains stable for eight more iterations before beginning to drop around iteration 15, likely because, at that point, the number of positive documents remaining in the AL Pool has decreased enough that they are more difficult to find. To better understand the contribution of different features to performance across iterations, we created ranked lists of the highest information gain features at each of the 20 classification tasks. Information gain was calculated according to

(6) $$I(X|Y)=\sum_{y\epsilon Y}\sum_{x\epsilon X}p(x,\;y)\log\left(\frac{p(x,\;y)}{p(x)p(y)}\right),$$

where *p*(*x*, *y*) is the joint probability distribution of *X* and *Y*, and *p*(*x*) and *p*(*y*) are the marginal probability distribution functions of *X* and *Y*, respectively. The top 20 information gain-scoring features at each iteration of the AL procedure are shown in Figure 7, where the word in the first position is the one that was the most informative for classification on that particular round, and its color corresponds to which part of the document meta data it came from (either title (black), abstract (red), or MeSH term (blue)). Although information gain is not used by SVMs for classification, it is a way to indirectly measure which features are, in general, most informative for distinguishing the two classes. As the figure shows, the predictive features used at the first iteration of AL are a mix of cell-related terms (e.g., *ganglion*), relations (e.g., *expressed*), and stop words (e.g., *at*, or *than*). After the first iteration, stop words (defined here as terms unrelated to biological research, such as *at*, and *in*) are hardly used, and more cell-related terminology begins to show up. By the final iteration, a stable selection of features has been identified, coming from the title, abstract, and MeSH terms. *Patch-clamp*, for example, is a method used to study ion channels in the cell, and could be used to collect data for a variety of types of NR submissions. The presence of *ganglion* (likely, from dorsal root ganglion cell) and *purkinje* (from purkinje cell) are not surprising either—both of these cell types have been extensively studied in the literature.

## 4. Discussion

The creation and maintenance of machine-readable public repositories of scientific information is an increasingly important and interesting area of informatics research. In this work, we demonstrate the utility of AL systems for aiding in the community-curated database biocuration workflow. Using a simple approach, incorporating binary feature modeling of documents' title, abstract, and MeSH terms, and an SVM classifier, we developed a system that shows how AL can be used by biocurators to quickly add annotated resources to an under-developed database while simultaneously training an ML classifier for later large-scale use. For completeness, here, we assumed that no previously-curated data was available for the initial training data. It's important to note that, for many knowledge bases, this would not be the case—some small amount of seed training data may already be available to biocurators, making the start-up costs of using our approach quite small.

Our research made several important contributions to the NR, as well as to the neuroinformatics community at-large, by improving several aspects of the NR knowledge base—we increased the number of entries by a factor of 90%, we increased its coverage of terms from the NeuroLex ontology, and we created an expert-curated neuroscience document collection that has been manually annotated according to whether each contains information that is relevant to the NR (i.e., it contains curatable information about neuron-related phenomena). According to our calculations, based on annotation rates observed in this study, we were able to make approximately 1–2 years worth of annotation contributions to the NR in only 4 months. The first two of these contributions will help make the NR into a resource that can be used by multi-level modelers in neuroinformatics (e.g., researchers interested in integrating information across multiple levels of the brain), as well as researchers wishing to find cited information on the central nervous system at the neuron level. By improving the coverage and depth of the NR, we have helped increase the visibility of the NR, and therefore increase the likelihood that it will obtain new users and experts who will be willing to add their own contributions to the database.

Our approach is similar to that of weakly supervised learning (e.g., Zhang, 2004), which works to improve classifier accuracy by adding documents about which the classifier is certain to the training model. In contrast to this approach, which adds these documents without the oversight of an expert reviewer, our system requests the expert gold-standard input on a set of documents about which the classifier is least certain. There were several reasons our approach was successful here. First, our corpus was selected because it was likely to contain information relevant to the Neuron Registry knowledge base. This increased the likelihood that any relevant documents were found in the initial random sample for the baseline training data. Had no positive-class samples been found, the classifier would not have had a starting point for how to identify relevant documents. Second, the annotator had a graduate level education in Neuroscience, this allowed for efficient determination of the true class of each document. Just as important was the fact that the expert annotator used an *a priori*-defined annotation criteria, developed in conjunction with the Neuron Registry staff. Consistent expert decisions allowed the classifier to correctly create a model of the positive-class documents. Finally, we used a simple classification approach, based on features that were present in every record used for classification (i.e., title, abstract, and MeSH terms). Using more complex feature types, such as named entity recognition-based features, would have likely led to certain documents having empty feature sets, giving the classifier less information to work with. For example, it is feasible that a neuroscientist could write about neuroanatomy in such a way that would not be recognized by simple methods for detecting neuroanatomical terms. As we demonstrated in our follow-up experiments, there are a variety of ways for writing about the brain, and developing optimal methods for detecting mentions of neuroanatomical terms is, itself, its own active area of ML research (e.g., French, 2012).

The 962-document manually-curated collection that was created to run our experiments is also an important and useful contribution. We have released these data as open source on github, with the hope that other ML scientists can use it to train document classifiers for the neurosciences^11^ (the document corpus has also been registered as a resource with the Neuroscience Information Framework^12^). As the neuroscience literature base continues to grow, methods for information retrieval and information extraction will continue to increase in importance, to neuroscientists and neuroinformaticians alike. Many such resources are trained on expertly-curated document collections that can be expensive to obtain. Here, we created a large collection of documents for training supervised algorithms. Our document collection has been annotated at both the document (i.e., relevant vs. irrelevant) and sentence level (the sentence(s) containing the information that led to a *relevant* judgment), so, in addition to being useful for training document classification systems, it could be used for training structure classification algorithms for information extraction. No such resource was previously available for neuroinformatics.

One limitation of the presently-described experiments is that only one curator (KHA) was used to assign inclusion/exclusion judgments to documents in the seed training collection, AL pool, and evaluation collection. Although the curator has a graduate-level education in the neurosciences, and the annotation criteria used was developed in collaboration with a professional biocurators and the NR staff, it would have been preferable to use a team of curators to assign labels, so that inter-annotator agreement statistics could have been calculated. Despite this limitation, all the annotations submitted over the course of this study have been reviewed and accepted by the NR curation staff. Along similar lines, in order to simplify the full text document acquisition step of our experiments, we limited our training data collection to only articles published in 2010, in six top neuroscience journals. We made this choice in order to ensure that adequate metadata would be available in MEDLINE, and to maximize the number of articles that we would encounter that would be relevant to the NR, while still being true to the diversity of the neuroscience literature base as a whole. Although this wasn't an especially limiting assumption, future work could extend that presented here by taking a larger, more chronologically diverse slice of neuroscience publications. This would enable future work to look at the role of concept drift (e.g., Forman, 2006) in the performance of AL and recommender systems for biocuration. Because our target knowledge base was specifically concerned with neuron-related information, we acquired a document corpus that reflected this focus. Although the *Virk* system is not meant to only be used for neuroscience use cases, future work should demonstrate that our approach will generalize to knowledge bases in other domains. Our system was intentionally designed with simple input feature types to the classifier (e.g., simple unigrams, rather than knowledge-engineering-based approaches, such as named-entity recognition). As in Ambert and Cohen (2011), it is likely that there are aspects of our pipeline that can and should be optimized for particular domains. Here, we have demonstrated the baseline efficacy of our general framework, using data set of neuroscience-related documents as an example use case. We evaluated an alternative, more complex feature modeling approach just to provide a reference point to the baseline simple feature modeling approach, and to show that no drastic performance benefits were being missed in our simple approach. A possible limitation of the current work is the manner in which mutual information-based feature modeling was carried out. The performance of our baseline classifier using this simple mutual information calculation was sufficient that we did not optimize it for differences in class sizes, which could have held back performance somewhat. An interesting future avenue for future experiments is determining the role of feature modeling algorithms in the performance of an AL system. Finally, future research could use other classification approaches beyond SVM. Although, as noted above, SVMs are known to perform well on textual data, it is possible that others may provide similarly good performance. Future work should investigate the relationship between input features, feature modeling techniques, and approaches to classification. We hope to motivate such work by making our data set available to researchers. One motivation for using an SVM classifier for the present set of experiments is that SVMs are very fast at classifying new documents. Training them, however, can take some time, and typically yields a squared-increase in training time, with the number of input documents. For our small use case of bootstrapping the start-up of a knowledge base, here, this was not a problem. However, for larger training corpuses, this might become an issue for biocuration workflows, and alternative classification approaches could be investigated. Researchers wishing to apply our approach to a new knowledge base should consider that there is going to be some start-up costs associated with training a classification system for their domain. A benefit of our approach is that, in terms of time spent, much of the start-up costs are associated with reading publications to determine which contain relevant information—something that would need to be done to create the knowledge base anyway.

Similar to others (Ambert and Cohen, 2009; Cohen et al., 2009, 2010b; Kouznetsov et al., 2009; Krallinger et al., 2009; Uzuner, 2009; Wallace et al., 2010), we used AUC and ranking to evaluate the system and prioritize the literature for annotation work. Another potential area for future research lies in optimizing the number of uncertain documents used to train the classifier at each iteration (here, 10 documents) and the number of predicted-relevant documents used for annotation and evaluation (here, 20 documents). Our values were selected as practical heuristics, based on what we thought the smallest number of documents we could review during each round of annotation that would yield usable confidence intervals. Future work could explore the effect of changing these values, and how the performance they yield interacts with characteristics of the corpus, such as the number of relevant documents thought to be contained in it.

The results of our experiment comparing a system trained with only the uncertain documents to one trained on all the available documents are intriguing. While the system trained on all the available annotated documents consistently outperformed the system trained only on the uncertain documents in terms of AUC, this comparison is incomplete. An important part of our system is the method used to select the most informative documents for manual annotation and addition to the training set in the next iteration. In our system, we used the simple approach of choosing the 10 documents for which the classifier had the most uncertainty—the documents with the lowest confidence in their predictions. This enabled us to keep the prevalence of the training set approximately equal to that of the document pool. If we had included all of the annotated documents in each round of training (both the uncertain and confident documents), the training set would become gradually more and more skewed toward the positive documents and therefore our simple approach of selecting the documents with the most uncertainty would also be subject to this bias. It is unclear what the impact would be on the performance of our system in this situation. More sophisticated means of choosing the most informative documents could avoid this problem and allow training on all of the annotated documents at each stage without risk of biasing the classifier. However, these methods tend to be much more computationally and algorithmically complex than the simple method that was effective here (Tong and Koller, 2002; Settles, 2010; Figueroa et al., 2012).

Another possibility is to start off training on only the annotated uncertain documents and after some number of iterations switch to including all of the annotated documents. An avenue for future investigation will be to examine *a priori* methods for identifying the point at which this switch should be made-in our studies, based on Figure 3, it appeared that this point occurred somewhere between iterations 10 and 14, but this may have been influenced by some aspects of our experiments (e.g., class distribution, or the number of documents used from training at each iteration). Finally, although our system is adept at expanding an online knowledge base (one bottleneck in the workflow of a community-curated database), it does nothing to address other inefficiencies, such as identifying likely erroneous submissions, recognizing newly-published articles that contain information of interest, or identifying where in an article the annotatable information could be found. Each of these, however, should be points of focus for future work.

### Conflict of interest statement

The authors declare that the research was conducted in the absence of any commercial or financial relationships that could be construed as a potential conflict of interest.

## Footnotes

^1^http://flybase.org/.

^2^http://wheat.pw.usda.gov/GG2/index.shtml.

^3^http://cocomac.org.

^4^http://pons.neurocommons.org/page/Neuron_registry.

^5^http://incfnrci.appspot.com/.

^6^http://www.neurolex.org.

^7^http://www.ncbi.nlm.nih.gov/pubmed/.

^8^www.mongodb.org.

^9^http://neurolex.org/wiki/Neuron_Types_With_Definitions.

^10^http://neurolex.org.

^11^https://github.com/ambertk/NeuronRegistryCorpus.

^12^http://www.neuinfo.org/.
